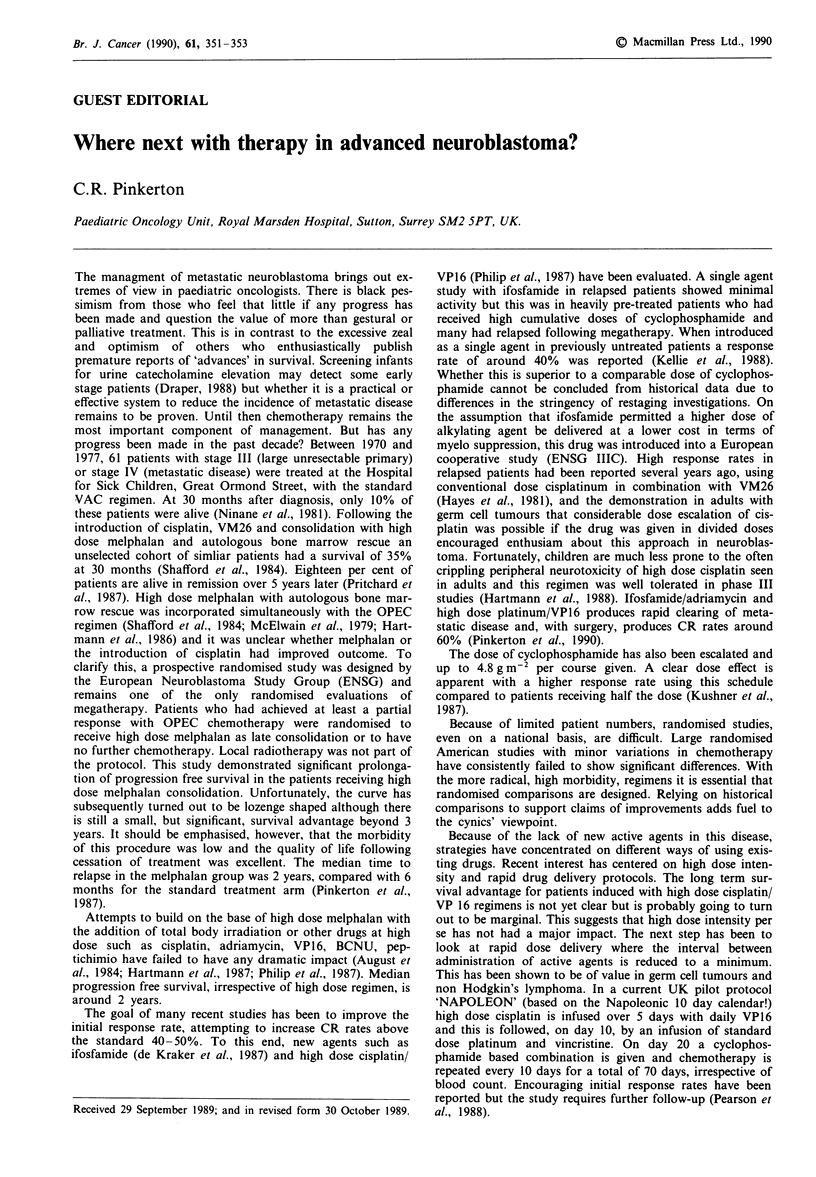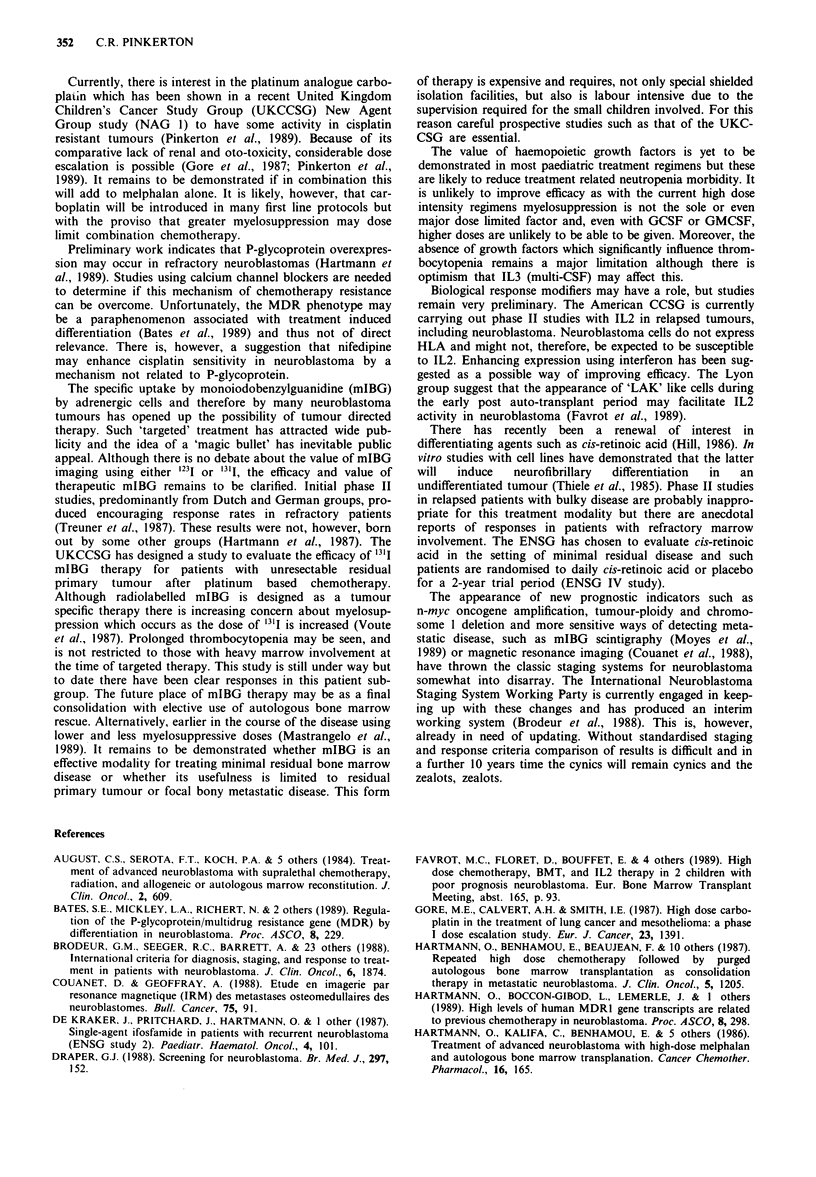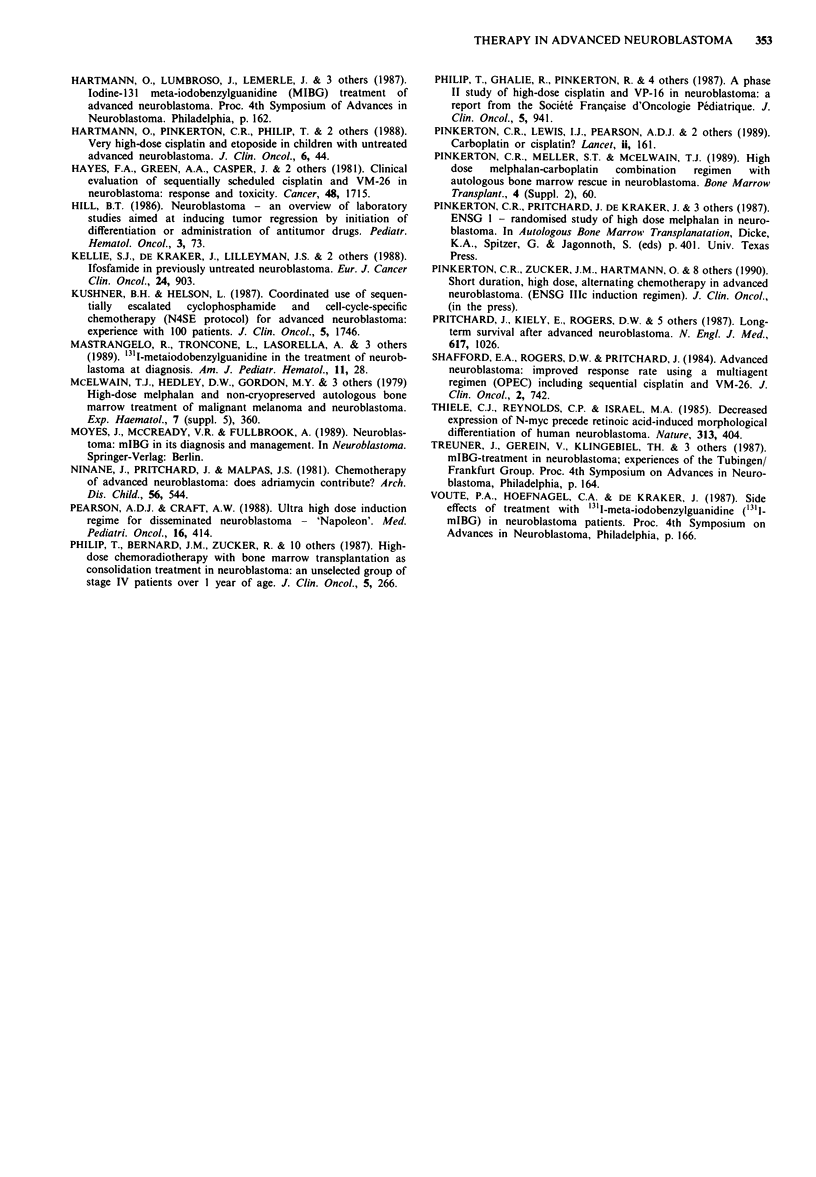# Where next with therapy in advanced neuroblastoma?

**DOI:** 10.1038/bjc.1990.77

**Published:** 1990-03

**Authors:** C. R. Pinkerton

**Affiliations:** Paediatric Oncology Unit, Royal Marsden Hospital, Sutton, Surrey, UK.


 © Macmillan Press Ltd., 1990

GUEST EDITORIAL

### Where next with therapy in advanced neuroblastoma?

C.R. Pinkerton

*Paediatric Oncology Unit, Royal Marsden Hospital, Sutton, Surrey SM2 5PT, UK.*

The managment of metastatic neuroblastoma brings out extremes of view in paediatric oncologists. There is black pessimism from those who feel that little if any progress has been made and question the value of more than gestural or palliative treatment. This is in contrast to the excessive zeal and optimism of others who enthusiastically publish premature reports of 'advances' in survival. Screening infants for urine catecholamine elevation may detect some early stage patients (Draper, 1988) but whether it is a practical or effective system to reduce the incidence of metastatic disease remains to be proven. Until then chemotherapy remains the most important component of management. But has any progress been made in the past decade? Between 1970 and 1977, 61 patients with stage III (large unresectable primary) or stage IV (metastatic disease) were treated at the Hospital for Sick Children, Great Ormond Street, with the standard VAC regimen. At 30 months after diagnosis, only 10% of these patients were alive (Ninane *et al.*, 1981). Following the introduction of cisplatin, VM26 and consolidation with high dose melphalan and autologous bone marrow rescue an unselected cohort of simliar patients had a survival of 35% at 30 months (Shafford *et al.*, 1984). Eighteen per cent of patients are alive in remission over 5 years later (Pritchard *et al.*, 1987). High dose melphalan with autologous bone marrow rescue was incorporated simultaneously with the OPEC regimen (Shafford *et al.*, 1984; McElwain *et al.*, 1979; Hartmann *et al.*, 1986) and it was unclear whether melphalan or the introduction of cisplatin had improved outcome. To clarify this, a prospective randomised study was designed by the European Neuroblastoma Study Group (ENSG) and remains one of the only randomised evaluations of megatherapy. Patients who had achieved at least a partial response with OPEC chemotherapy were randomised to receive high dose melphalan as late consolidation or to have no further chemotherapy. Local radiotherapy was not part of the protocol. This study demonstrated significant prolongation of progression free survival in the patients receiving high dose melphalan consolidation. Unfortunately, the curve has subsequently turned out to be lozenge shaped although there is still a small, but significant, survival advantage beyond 3 years. It should be emphasised, however, that the morbidity of this procedure was low and the quality of life following cessation of treatment was excellent. The median time to relapse in the melphalan group was 2 years, compared with 6 months for the standard treatment arm (Pinkerton *et al.*, 1987).

Attempts to build on the base of high dose melphalan with the addition of total body irradiation or other drugs at high dose such as cisplatin, adriamycin, VP16, BCNU, peptichimio have failed to have any dramatic impact (August *et al.*, 1984; Hartmann *et al.*, 1987; Philip *et al.*, 1987). Median progression free survival, irrespective of high dose regimen, is around 2 years.

The goal of many recent studies has been to improve the initial response rate, attempting to increase CR rates above the standard 40–50%. To this end, new agents such as ifosfamide (de Kraker *et al.*, 1987) and high dose cisplatin/VP16 (Philip *et al.*, 1987) have been evaluated. A single agent study with ifosfamide in relapsed patients showed minimal activity but this was in heavily pre-treated patients who had received high cumulative doses of cyclophosphamide and many had relapsed following megatherapy. When introduced as a single agent in previously untreated patients a response rate of around 40% was reported (Kellie *et al.*, 1988). Whether this is superior to a comparable dose of cyclophosphamide cannot be concluded from historical data due to differences in the stringency of restaging investigations. On the assumption that ifosfamide permitted a higher dose of alkylating agent be delivered at a lower cost in terms of myelo suppression, this drug was introduced into a European cooperative study (ENSG IIIC). High response rates in relapsed patients had been reported several years ago, using conventional dose cisplatinum in combination with VM26 (Hayes *et al.*, 1981), and the demonstration in adults with germ cell tumours that considerable dose escalation of cisplatin was possible if the drug was given in divided doses encouraged enthusiam about this approach in neuroblastoma. Fortunately, children are much less prone to the often crippling peripheral neurotoxicity of high dose cisplatin seen in adults and this regimen was well tolerated in phase III studies (Hartmann *et al.*, 1988). Ifosfamide/adriamycin and high dose platinum/VP16 produces rapid clearing of metastatic disease and, with surgery, produces CR rates around 60% (Pinkerton *et al.*, 1990).

The dose of cyclophosphamide has also been escalated and up to $4.8\,g\,m^{-2}$ per course given. A clear dose effect is apparent with a higher response rate using this schedule compared to patients receiving half the dose (Kushner *et al.*, 1987).

Because of limited patient numbers, randomised studies, even on a national basis, are difficult. Large randomised American studies with minor variations in chemotherapy have consistently failed to show significant differences. With the more radical, high morbidity, regimens it is essential that randomised comparisons are designed. Relying on historical comparisons to support claims of improvements adds fuel to the cynics' viewpoint.

Because of the lack of new active agents in this disease, strategies have concentrated on different ways of using existing drugs. Recent interest has centered on high dose intensity and rapid drug delivery protocols. The long term survival advantage for patients induced with high dose cisplatin/VP 16 regimens is not yet clear but is probably going to turn out to be marginal. This suggests that high dose intensity per se has not had a major impact. The next step has been to look at rapid dose delivery where the interval between administration of active agents is reduced to a minimum. This has been shown to be of value in germ cell tumours and non Hodgkin's lymphoma. In a current UK pilot protocol 'NAPOLEON' (based on the Napoleonic 10 day calendar!) high dose cisplatin is infused over 5 days with daily VP16 and this is followed, on day 10, by an infusion of standard dose platinum and vincristine. On day 20 a cyclophosphamide based combination is given and chemotherapy is repeated every 10 days for a total of 70 days, irrespective of blood count. Encouraging initial response rates have been reported but the study requires further follow-up (Pearson *et al.*, 1988).

Received 29 September 1989; and in revised form 30 October 1989.

Currently, there is interest in the platinum analogue carboplatin which has been shown in a recent United Kingdom Children's Cancer Study Group (UKCCSG) New Agent Group study (NAG 1) to have some activity in cisplatin resistant tumours (Pinkerton *et al.*, 1989). Because of its comparative lack of renal and oto-toxicity, considerable dose escalation is possible (Gore *et al.*, 1987; Pinkerton *et al.*, 1989). It remains to be demonstrated if in combination this will add to melphalan alone. It is likely, however, that carboplatin will be introduced in many first line protocols but with the proviso that greater myelosuppression may dose limit combination chemotherapy.

Preliminary work indicates that P-glycoprotein overexpression may occur in refractory neuroblastomas (Hartmann *et al.*, 1989). Studies using calcium channel blockers are needed to determine if this mechanism of chemotherapy resistance can be overcome. Unfortunately, the MDR phenotype may be a paraphenomenon associated with treatment induced differentiation (Bates *et al.*, 1989) and thus not of direct relevance. There is, however, a suggestion that nifedipine may enhance cisplatin sensitivity in neuroblastoma by a mechanism not related to P-glycoprotein.

The specific uptake by monoiodobenzylguanidine (mIBG) by adrenergic cells and therefore by many neuroblastoma tumours has opened up the possibility of tumour directed therapy. Such 'targeted' treatment has attracted wide publicity and the idea of a 'magic bullet' has inevitable public appeal. Although there is no debate about the value of mIBG imaging using either $^{123}I$ or $^{131}I$, the efficacy and value of therapeutic mIBG remains to be clarified. Initial phase II studies, predominantly from Dutch and German groups, produced encouraging response rates in refractory patients (Treuner *et al.*, 1987). These results were not, however, born out by some other groups (Hartmann *et al.*, 1987). The UKCCSG has designed a study to evaluate the efficacy of $^{131}I$ mIBG therapy for patients with unresectable residual primary tumour after platinum based chemotherapy. Although radiolabelled mIBG is designed as a tumour specific therapy there is increasing concern about myelosuppression which occurs as the dose of $^{131}I$ is increased (Voute *et al.*, 1987). Prolonged thrombocytopenia may be seen, and is not restricted to those with heavy marrow involvement at the time of targeted therapy. This study is still under way but to date there have been clear responses in this patient subgroup. The future place of mIBG therapy may be as a final consolidation with elective use of autologous bone marrow rescue. Alternatively, earlier in the course of the disease using lower and less myelosuppressive doses (Mastrangelo *et al.*, 1989). It remains to be demonstrated whether mIBG is an effective modality for treating minimal residual bone marrow disease or whether its usefulness is limited to residual primary tumour or focal bony metastatic disease. This form of therapy is expensive and requires, not only special shielded isolation facilities, but also is labour intensive due to the supervision required for the small children involved. For this reason careful prospective studies such as that of the UKCCSG are essential.

The value of haemopoietic growth factors is yet to be demonstrated in most paediatric treatment regimens but these are likely to reduce treatment related neutropenia morbidity. It is unlikely to improve efficacy as with the current high dose intensity regimens myelosuppression is not the sole or even major dose limited factor and, even with GCSF or GMCSF, higher doses are unlikely to be able to be given. Moreover, the absence of growth factors which significantly influence thrombocytopenia remains a major limitation although there is optimism that IL3 (multi-CSF) may affect this.

Biological response modifiers may have a role, but studies remain very preliminary. The American CCSG is currently carrying out phase II studies with IL2 in relapsed tumours, including neuroblastoma. Neuroblastoma cells do not express HLA and might not, therefore, be expected to be susceptible to IL2. Enhancing expression using interferon has been suggested as a possible way of improving efficacy. The Lyon group suggest that the appearance of 'LAK' like cells during the early post auto-transplant period may facilitate IL2 activity in neuroblastoma (Favrot *et al.*, 1989).

There has recently been a renewal of interest in differentiating agents such as *cis*-retinoic acid (Hill, 1986). *In vitro* studies with cell lines have demonstrated that the latter will induce neurofibrillary differentiation in an undifferentiated tumour (Thiele *et al.*, 1985). Phase II studies in relapsed patients with bulky disease are probably inappropriate for this treatment modality but there are anecdotal reports of responses in patients with refractory marrow involvement. The ENSG has chosen to evaluate *cis*-retinoic acid in the setting of minimal residual disease and such patients are randomised to daily *cis*-retinoic acid or placebo for a 2-year trial period (ENSG IV study).

The appearance of new prognostic indicators such as n-*myc* oncogene amplification, tumour-ploidy and chromosome 1 deletion and more sensitive ways of detecting metastatic disease, such as mIBG scintigraphy (Moyes *et al.*, 1989) or magnetic resonance imaging (Couanet *et al.*, 1988), have thrown the classic staging systems for neuroblastoma somewhat into disarray. The International Neuroblastoma Staging System Working Party is currently engaged in keeping up with these changes and has produced an interim working system (Brodeur *et al.*, 1988). This is, however, already in need of updating. Without standardised staging and response criteria comparison of results is difficult and in a further 10 years time the cynics will remain cynics and the zealots, zealots.

### References

AUGUST, C.S., SEROTA, F.T., KOCH, P.A. & 5 others (1984). Treatment of advanced neuroblastoma with supralethal chemotherapy, radiation, and allogeneic or autologous marrow reconstitution. *J. Clin. Oncol.*, **2**, 609.

BATES, S.E., MICKLEY, L.A., RICHERT, N. & 2 others (1989). Regulation of the P-glycoprotein/multidrug resistance gene (MDR) by differentiation in neuroblastoma. *Proc. ASCO*, **8**, 229.

BRODEUR, G.M., SEEGER, R.C., BARRETT, A. & 23 others (1988). International criteria for diagnosis, staging, and response to treatment in patients with neuroblastoma. *J. Clin. Oncol.*, **6**, 1874.

COUANET, D. & GEOFFRAY, A. (1988). Etude en imagerie par resonance magnetique (IRM) des metastases osteomedullaires des neuroblastomes. *Bull. Cancer*, **75**, 91.

DE KRAKER, J., PRITCHARD, J., HARTMANN, O. & 1 other (1987). Single-agent ifosfamide in patients with recurrent neuroblastoma (ENSG study 2). *Paediatr. Haematol. Oncol.*, **4**, 101.

DRAPER, G.J. (1988). Screening for neuroblastoma. *Br. Med. J.*, **297**, 152.

FAVROT, M.C., FLORET, D., BOUFFET, E. & 4 others (1989). High dose chemotherapy, BMT, and IL2 therapy in 2 children with poor prognosis neuroblastoma. Eur. Bone Marrow Transplant Meeting, abst. 165, p. 93.

GORE, M.E., CALVERT, A.H. & SMITH, I.E. (1987). High dose carboplatin in the treatment of lung cancer and mesothelioma: a phase I dose escalation study. *Eur. J. Cancer*, **23**, 1391.

HARTMANN, O., BENHAMOU, E., BEAUJEAN, F. & 10 others (1987). Repeated high dose chemotherapy followed by purged autologous bone marrow transplantation as consolidation therapy in metastatic neuroblastoma. *J. Clin. Oncol.*, **5**, 1205.

HARTMANN, O., BOCCON-GIBOD, L., LEMERLE, J. & 1 others (1989). High levels of human MDR1 gene transcripts are related to previous chemotherapy in neuroblastoma. *Proc. ASCO*, **8**, 298.

HARTMANN, O., KALIFA, C., BENHAMOU, E. & 5 others (1986). Treatment of advanced neuroblastoma with high-dose melphalan and autologous bone marrow transplanation. *Cancer Chemother. Pharmacol.*, **16**, 165.

HARTMANN, O., LUMBROSO, J., LEMERLE, J. & 3 others (1987). Iodine-131 meta-iodobenzylguanidine (MIBG) treatment of advanced neuroblastoma. Proc. 4th Symposium of Advances in Neuroblastoma. Philadelphia, p. 162.

HARTMANN, O., PINKERTON, C.R., PHILIP, T. & 2 others (1988). Very high-dose cisplatin and etoposide in children with untreated advanced neuroblastoma. *J. Clin. Oncol.*, **6**, 44.

HAYES, F.A., GREEN, A.A., CASPER, J. & 2 others (1981). Clinical evaluation of sequentially scheduled cisplatin and VM-26 in neuroblastoma: response and toxicity. *Cancer*, **48**, 1715.

HILL, B.T. (1986). Neuroblastoma - an overview of laboratory studies aimed at inducing tumor regression by initiation of differentiation or administration of antitumor drugs. *Pediatr. Hematol. Oncol.*, **3**, 73.

KELLIE, S.J., DE KRAKER, J., LILLEYMAN, J.S. & 2 others (1988). Ifosfamide in previously untreated neuroblastoma. *Eur. J. Cancer Clin. Oncol.*, **24**, 903.

KUSHNER, B.H. & HELSON, L. (1987). Coordinated use of sequentially escalated cyclophosphamide and cell-cycle-specific chemotherapy (N4SE protocol) for advanced neuroblastoma: experience with 100 patients. *J. Clin. Oncol.*, **5**, 1746.

MASTRANGELO, R., TRONCONE, L., LASORELLA, A. & 3 others (1989). $^{131}$I-metaiodobenzylguanidine in the treatment of neuroblastoma at diagnosis. *Am. J. Pediatr. Hematol.*, **11**, 28.

McELWAIN, T.J., HEDLEY, D.W., GORDON, M.Y. & 3 others (1979) High-dose melphalan and non-cryopreserved autologous bone marrow treatment of malignant melanoma and neuroblastoma. *Exp. Haematol.*, **7** (suppl. 5), 360.

MOYES, J., MCCREADY, V.R. & FULLBROOK, A. (1989). Neuroblastoma: mIBG in its diagnosis and management. In *Neuroblastoma.* Springer-Verlag: Berlin.

NINANE, J., PRITCHARD, J. & MALPAS, J.S. (1981). Chemotherapy of advanced neuroblastoma: does adriamycin contribute? *Arch. Dis. Child.*, **56**, 544.

PEARSON, A.D.J. & CRAFT, A.W. (1988). Ultra high dose induction regime for disseminated neuroblastoma - 'Napoleon'. *Med. Pediatri. Oncol.*, **16**, 414.

PHILIP, T., BERNARD, J.M., ZUCKER, R. & 10 others (1987). High-dose chemoradiotherapy with bone marrow transplantation as consolidation treatment in neuroblastoma: an unselected group of stage IV patients over 1 year of age. *J. Clin. Oncol.*, **5**, 266.

PHILIP, T., GHALIE, R., PINKERTON, R. & 4 others (1987). A phase II study of high-dose cisplatin and VP-16 in neuroblastoma: a report from the Société Française d'Oncologie Pédiatrique. *J. Clin. Oncol.*, **5**, 941.

PINKERTON, C.R., LEWIS, I.J., PEARSON, A.D.J. & 2 others (1989). Carboplatin or cisplatin? *Lancet*, **ii**, 161.

PINKERTON, C.R., MELLER, S.T. & McELWAIN, T.J. (1989). High dose melphalan-carboplatin combination regimen with autologous bone marrow rescue in neuroblastoma. *Bone Marrow Transplant.*, **4** (Suppl. 2), 60.

PINKERTON, C.R., PRITCHARD, J. DE KRAKER, J. & 3 others (1987). ENSG 1 - randomised study of high dose melphalan in neuroblastoma. In *Autologous Bone Marrow Transplanatation*, Dicke, K.A., Spitzer, G. & Jagonnoth, S. (eds) p. 401. Univ. Texas Press.

PINKERTON, C.R., ZUCKER, J.M., HARTMANN, O. & 8 others (1990). Short duration, high dose, alternating chemotherapy in advanced neuroblastoma. (ENSG IIIc induction regimen). *J. Clin. Oncol.*, (in the press).

PRITCHARD, J., KIELY, E., ROGERS, D.W. & 5 others (1987). Long-term survival after advanced neuroblastoma. *N. Engl. J. Med.*, **617**, 1026.

SHAFFORD, E.A., ROGERS, D.W. & PRITCHARD, J. (1984). Advanced neuroblastoma: improved response rate using a multiagent regimen (OPEC) including sequential cisplatin and VM-26. *J. Clin. Oncol.*, **2**, 742.

THIELE, C.J., REYNOLDS, C.P. & ISRAEL, M.A. (1985). Decreased expression of N-myc precede retinoic acid-induced morphological differentiation of human neuroblastoma. *Nature*, **313**, 404.

TREUNER, J., GEREIN, V., KLINGEBIEL, TH. & 3 others (1987). mIBG-treatment in neuroblastoma; experiences of the Tubingen/ Frankfurt Group. Proc. 4th Symposium on Advances in Neuroblastoma, Philadelphia, p. 164.

VOUTE, P.A., HOEFNAGEL, C.A. & DE KRAKER, J. (1987). Side effects of treatment with $^{131}$I-meta-iodobenzylguanidine ($^{131}$I-mIBG) in neuroblastoma patients. Proc. 4th Symposium on Advances in Neuroblastoma, Philadelphia, p. 166.